# Optogenetic Tractography for anatomo-functional characterization of cortico-subcortical neural circuits in non-human primates

**DOI:** 10.1038/s41598-018-21486-8

**Published:** 2018-02-20

**Authors:** S. Senova, C. Poupon, J. Dauguet, H. J. Stewart, G. P. Dugué, C. Jan, K. Hosomi, G. S. Ralph, L. Barnes, X. Drouot, C. Pouzat, J. F. Mangin, F. Pain, I. Doignon, R. Aron-Badin, E. Brouillet, E. S. Boyden, K. A. Mitrophanous, P. Hantraye, S. Palfi

**Affiliations:** 1 Laboratoire des Maladies Neurodégénératives, UMR9199, CEA, CNRS, Université Paris-Sud, Univ Paris Saclay, MIRCen, I²BM, CEA, Fontenay-aux-Roses, 92265 France; 2 Neurosurgery Department, Assistance Publique-Hôpitaux de Paris (APHP), Groupe Henri-Mondor Albert-Chenevier, PePsy department, F-94010 Créteil, France; 3 U955 INSERM IMRB eq. 14 Université Paris 12 UPEC, Faculté de Médecine, F-94010 Créteil, France; 4 Unité d’IRM et de Spectroscopie(UNIRS), NeuroSpin, I²BM, CEA, Saclay, France; 5 Oxford BioMedica (UK) Limited, Windrush Court, Transport Way, Oxford, UK; 6 CNRS UMR 8197, Inserm U1024, institut de Biologie de l’Ecole Normale Supérieure, Paris, France; 7 Functional Explorations department, Assistance Publique-Hôpitaux de Paris (APHP), Groupe Henri-Mondor Albert-Chenevier, Université Paris 12 UPEC, Faculté de Médecine, F-94010 Créteil, France; 8 MAP5, Mathématiques Appliquées Paris 5, CNRS UMR 8145 Paris, France; 9 Laboratoire de Neuroanatomie Assistée par Ordinateur (LNAO), CEA, Neurospin, Saclay, France; 10 IMNC-IN2P3, Université Paris Sud, Orsay, France; 11 INSERM UMRs 1174, Laboratory of Cellular interactions and liver physiopathology, Université Paris-Sud, Batiment 443, Orsay, France; 12 Massachusetts Institute of Technology, Media Lab, Boston, United States

## Abstract

Dissecting neural circuitry in non-human primates (NHP) is crucial to identify potential neuromodulation anatomical targets for the treatment of pharmacoresistant neuropsychiatric diseases by electrical neuromodulation. How targets of deep brain stimulation (DBS) and cortical targets of transcranial magnetic stimulation (TMS) compare and might complement one another is an important question. Combining optogenetics and tractography may enable anatomo-functional characterization of large brain cortico-subcortical neural pathways. For the proof-of-concept this approach was used in the NHP brain to characterize the motor cortico-subthalamic pathway (m_CSP) which might be involved in DBS action mechanism in Parkinson’s disease (PD). Rabies-G-pseudotyped and Rabies-G-VSVg-pseudotyped EIAV lentiviral vectors encoding the opsin *ChR2* gene were stereotaxically injected into the subthalamic nucleus (STN) and were retrogradely transported to the layer of the motor cortex projecting to STN. A precise anatomical mapping of this pathway was then performed using histology-guided high angular resolution MRI tractography guiding accurately cortical photostimulation of m_CSP origins. Photoexcitation of m_CSP axon terminals or m_CSP cortical origins modified the spikes distribution for photosensitive STN neurons firing rate in non-equivalent ways. Optogenetic tractography might help design preclinical neuromodulation studies in NHP models of neuropsychiatric disease choosing the most appropriate target for the tested hypothesis.

## Introduction

Designing electrical neuromodulation treatments that are more specific, efficient and therefore safer for neuropsychiatric diseases requires an accurate understanding of the brain anatomy and its relationship to function. The use of non-human primates (NHP) as a model to validate novel invasive therapeutic neuromodulation constitutes a necessary step prior to clinical application^1–4^. A better understanding of connectivity and functional differences between deep brain and potential cortical targets for neuromodulation is required. Indeed deep brain and cortical targets are now being investigated clinically worldwide with considerable success in a number of indications^5^. Here we describe a method for anatomo-functional characterisation of any cortico-subcortical fibre pathways with high precision and specificity in the large brain of the NHP.

*In vivo* precise and reliable morphological assessment of cortico-subcortical pathways in NHPs is challenging. Detailed mapping of brain connections with histological neural tracers is not compatible with *in vivo* neuromodulation. In addition mapping using electrical stimulation and *in vivo* recordings does not allow whole brain understanding of neural networks. In contrast, tractography reconstructions after *in vivo* diffusion weighted MRI acquisitions (DTI) is promising for this purpose^6,7^. However how these *in vivo* predictions compare to histological reality remains to be established especially when tracking sparse tracts^8–11^.

Accurate functional control of such pathways is complicated especially in large brains such as in NHPs and humans. Indeed brain electrical stimulation is limited by a lack of understanding at the level of the neuronal network and the use of pharmacological substances is not compatible with millisecond timescale neuromodulation^12^. In contrast, optogenetic technology enables a highly selective dissection of neural circuits^12–14^. While optogenetic has helped address important neurophysiological questions in rodent neurophysiology^15,16^, only modest behavioural responses to light activation have been obtained in NHPs thus far^14,17,18^. Among the major limitations are achieving efficient genetic modification of a large functionally coherent part of the cortex with the opsin gene while avoiding any deleterious cortical lesions following direct cortical injection of vectors^17^ and to shine sufficient light over the photosensitive brain volume to elicit a meaningful physiological response.

Thus we designed a 3-step strategy to address these challenges of anatomo-functional characterization of cortico-subcortical pathways in NHPs. First, to achieve a wide and comprehensive expression of the ChannelRhodopsin2 opsin (ChR2) in any functionally coherent large cortical area of the brain without directly altering it, we “remotely” delivered the eYFP/Channel Rhodopsin-2(ChR2) gene by injecting a lentiviral vector with retrograde transport properties into a smaller subcortical connected area of the NHP brain region that would constitute a “hub area of the brain”. We designed such a lentiviral vector enabling safe^19^, precise and spatially controlled retrograde opsin gene transfer in NHPs from the subthalamic nucleus (STN) back to the cortex. Adeno-associated virus (AAV) serotypes 6, 8 & 9 enable safe, diffuse and retrograde gene transfer, but they may diffuse too widely outside of small brain nuclei or display both anterograde and transynaptic gene transport that would compromise specificity^20^. Lentiviral vectors pseudotyped with the Rabies glycoprotein constitute an attractive alternative that is more suitable for precise retrograde transport as required in this study^21^. Second, we determined the optimal tractography reconstruction parameters for the motor cortico-subthalamic pathway (m_CSP) using HARDI (high angular resolution diffusion weighted imaging) acquisition by comparison to 3D-histological whole brain fluorescence maps. These parameters were determined for each STN-connected motor cortical region. Third, accurately guided by the predictions of tractography reconstructions after HARDI, we performed photo-excitation and functional characterization of both “ends” of the m_CSP.

As proof of concept, this method was used in normal NHPs to dissect the m_CSP, “the hyperdirect pathway”, suspected to be central in the physiopathology of Parkinson’s disease and its treatment by deep brain stimulation^15,22–27^.

## Results

### Step 1: Opsin transgenesis over a functionally coherent large cortical area in the non-human primate brain after retrograde gene transfer

**Figure 1:**
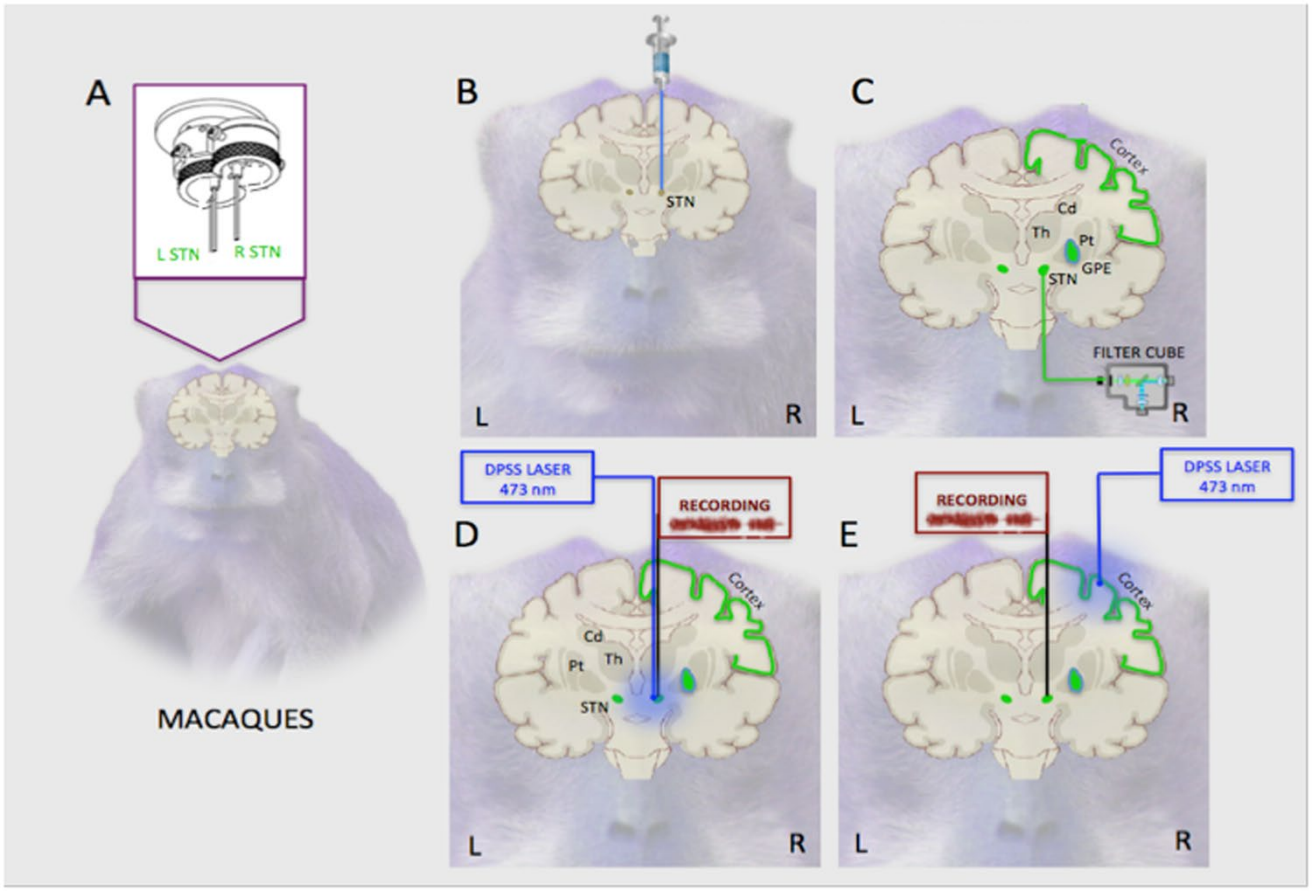
Experimental design. (**a)** At D0 a bilateral MRI-compatible chamber was chronically implanted above frontal cortex of NPH. Double channels MRI compatible cannulas were implanted ending typically 1 mm above the dorsolateral aspect of the left and/or right STN. (**b)** At D15, baseline fluorescence within STN was detected *in vivo* with a fibre-coupled minicube (NHP 3). Photostimulation above STN and electrophysiological recordings within the STN were performed as control experiments. Finally, an EIAV-Rabies-CaMK2-*ChR2-eYFP* lentiviral vector with retrograde transfer properties was injected unilaterally inside the STN. **(c)** At D75, *ChR2-eYFP* associated fluorescence was detected *in vivo* with a fibre-coupled minicube in STN (NHP 3), in order to confirm transduction (green). (**e)** At D75, in NHP 1 & 2, a fibre optics was lowered through one channel and set typically 1 mm above STN. An electrode was lowered through the other channel. The distance between the two channels main axes was 0.8 mm. High frequency blue photostimulation was performed above the dorsolateral STN, and STN neurons were recorded along a dorso-ventral axis. **(f)** At D75, in NHP 3, a fibre optics was lowered in the motor cortex through a craniotomy, guided by tractography reconstructions, and electrodes were lowered inside the ipsilateral STN through both channels. High frequency blue photostimulation was performed in the prmary motor cortex while STN neurons were recorded along a dorso-ventral axis at the same time. D: Day; MRI: magnetic resonance imaging; NHP: non-human primate; STN: subthalamic nucleus.

**Figure 2:**
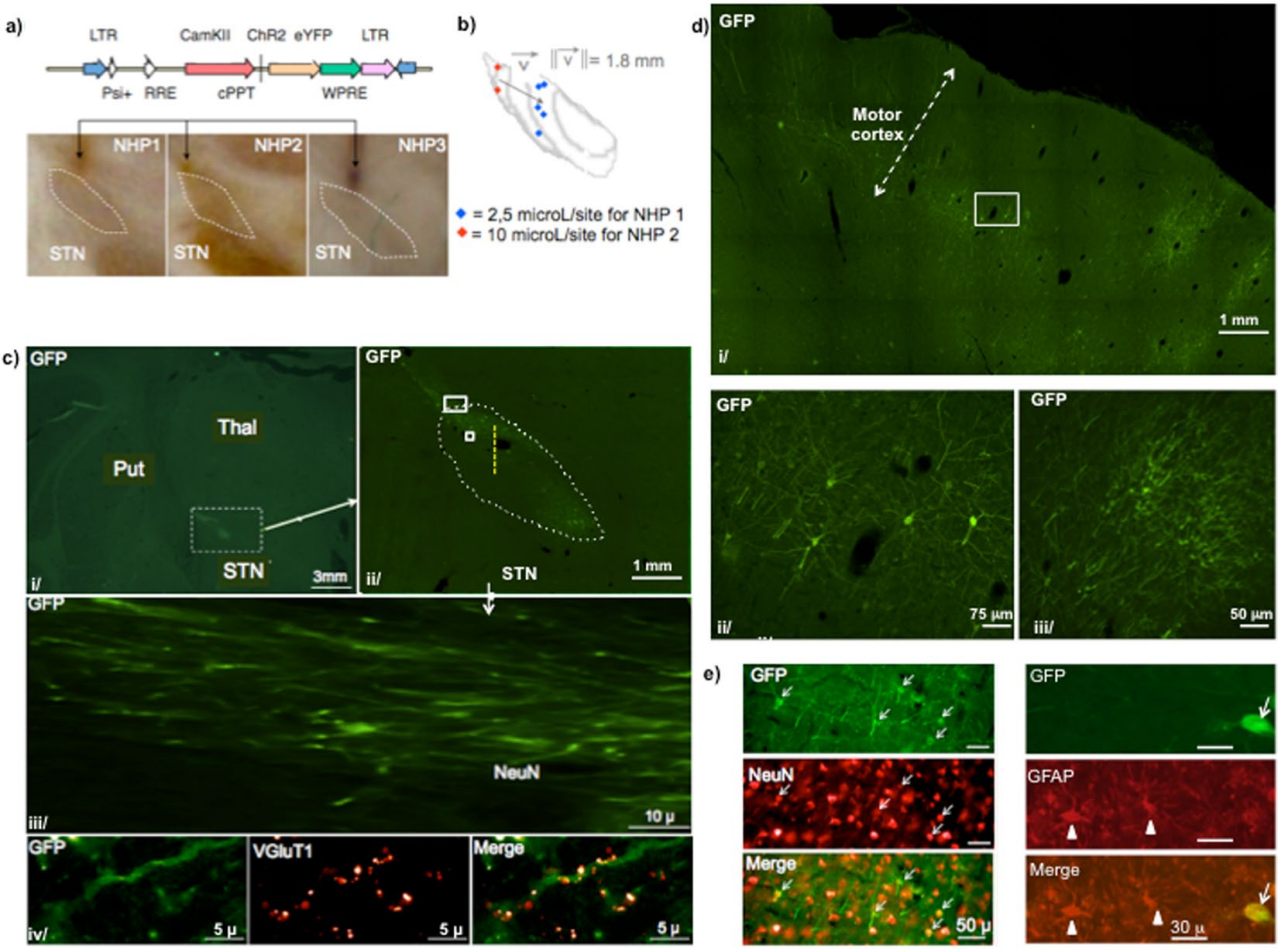
Histological characterization of the lentiviral vector enabling retrograde transport of C*hR2*. (**a)** EIAV-Rabies-CaMK2-*ChR2-eYFP* and EIAV-VSVg-Rabies-CaMK2-*ChR2-eYFP* lentiviral vectors with retrograde transfer properties were injected unilaterally in the STN of NHP 1 & 2, and NHP 3 respectively. Cannula traces were visualized on histological slices and confirmed to end above the dorsolateral aspect of STN of NHP 1, 2 & 3. (**b)** Injection sites for NHP 1 & 2 are 3D-reconstituted in a common STN after affine and elastic transformations between the two STN for NHP 1 & 2. (**c)** i/ high magnification on a coronal slice showing right STN, thalamus and putamen. ii/ white rectangle around STN on i/ is zoomed. White dots dlimit the STN. Yellow dashes indicate the recording electrode trajectory in the prolongation of the cannula. iii/ the biggest white rectangle in ii/ is zoomed: axons expressing *ChR2-eYFP* arriving to STN. iv/ the smallest white rectangle in ii/ is zoomed: VgluT1 positive cortico-subthalamic axon terminals expressing Ch*R2-eYFP*. (**d)** i/ zoom on a coronal motor cortex slice. ii/ white rectangle in i/ is zoomed: neurons of cortical layer V express *ChR2-eYFP* on their somas, dendrites and initial portion of their axons. iii/ dendrites expressing *ChR2-eYFP*. **(e)** Double immune-staining GFP/NeuN and GFP/GFAP in the layer V of motor cortex. White arrows indicate GFP-positive cells. White triangles indicate GFAP-positive cells NHP: Non-human primate; STN: subthalamic nucleus; Thal: thalamus; Put: putamen; VgluT1: vesicular glutamate transporter 1.

EIAV lentiviral vectors pseudotyped with Rabies G glycoprotein (NHP 1 & 2) or with a Rabies G glycoprotein and VSVg fusion protein (NHP 3) and coding for the expression of *ChR2-eYFP* gene under the control of the non-ubiquitous promoter CaMK2 (Figs 1, 2a) were produced, with a titer up to 8.1*10^7^ TU/ml (Figs 1 and 2).

Three months after lentiviral delivery to the STN, fluorescence was detected post-mortem in STN and in motor cortex. *ChR2-eYFP* was expressed on dendrites, somas and axons (Fig. 2c and d).

We used the promoter CaMK2, described as being selective for excitatory cortical neurons in NHPs^14,28^. The use of this promoter and an EIAV lentiviral vector ensured that vector expression was restricted to neuronal cells in transduced cortical regions (Fig. 2e).

Following retrograde gene transport, the number of motor cortex neurons expressing *ChR2-eYFP* was 4344 for NHP 1 and 10611 for NHP 2. Multiple optrode insertions within the motor cortex seriously damaged the brain tissue and prevented the quantification of transduced neurons in the cortex of NHP 3. Retrograde transfer to the centromedial thalamus was minimal (171 & 123 neurons for NHP 1 & 2) and absent in the pedunculopontine nucleus.

We did not detect any major sign of toxicity related to opsin expression in the cortex without cortical photostimulation (NHP 1&2) (Fig. 2e). NeuN and GFAP immune-staining were performed (Fig. 2e). Transduced cortical neurons displayed a normal shape. In a rat, we did not detect any sign of toxicity related to high frequency long-lasting blue photostimulation in the absence of vector injection (Supplementary Figure 1).

**Figure 3:**
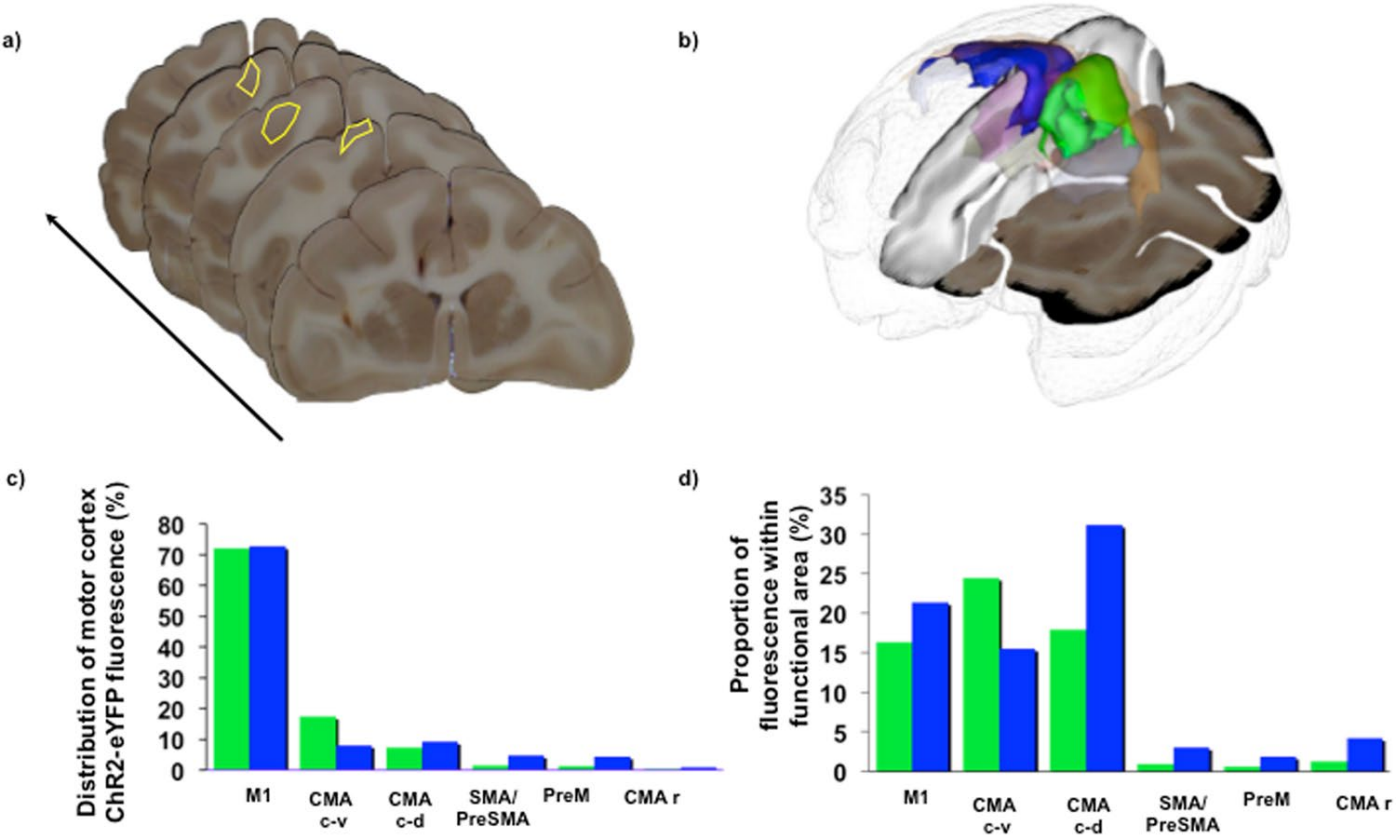
Three dimensional reconstruction of *ChR2-eYFP* associated fluorescence at the cortical origins of the motor-cortico-subthalamic tract. (**a)** Whole brains of NHP 1 & 2 were sliced. Every eight 40-µm thick coronal slice was photographed, and a whole brain histological block was reconstituted. *ChR2-eYFP* fluorescence detected with a microscope after immunohistochemistry was segmented on each slice. (**b)** *ChR2-eYFP* motor cortex fluorescence were 3D-reconstructed for NHP 1 (green) and NHP 2 (blue) and were expressed in a common space. (**c)** Six motor cortex functional regions were found to express fluorescence. Distribution of ChR2-eYFP fluorescence in the motor cortex was computed. (**d)** For each of these six regions, the proportion of the region expressing fluorescence was calculated. CMA c-v: caudo-ventral cingular motor cortex; CMA c-d: caudo-dorsal cingular motor cortex; CMAr: rostral cingular motor cortex; M1: primary motor cortex; NHP: non-human primate; PreM: premotor cortex; SMA-PreSMA: supplementary motor area-presupplementary motor area.

3D-histological reconstruction of the cortical volume in which fluorescence associated to *ChR2-eYFP* expression was detected by fluorescence microscopy (NHP 1&2) showed that retrograde transport occurred over distances up to 30 mm (Fig. 3a,b). Fluorescence was detected in the primary motor cortex (72.4 +/− 0.4% of whole ChR2-eYFP associated brain fluorescence), premotor cortex (2.82 +/− 2.2%), supplementary motor area (3.1 +/− 2.3%), caudo-ventral cingular cortex (12.7 +/− 6.6%), caudo-dorsal cingular cortex (8.3 +/− 1.3%) and rostral cingular cortex (0.7 +/− 0.3%) (Fig. 3c). Fluorescence was also detected in 18.9 +/− 3.6% of primary motor cortex volume, 1.2 +/− 0.9% of premotor cortex volume, 2 +/− 1.5% of supplementary motor area volume, 20 +/− 6.3% of caudo-ventral cingular cortex volume, 24.5 +/− 9.3% of caudo-dorsal cingular cortex volume and 2.7 +/− 2.1% of rostral cingular cortex volume (Fig. 3d). Motor cortex projections to the STN were originating from layer 5 (Fig. 2d). Fluorescence was detected within fibres of the pyramidal tract and some axon terminals in the striatum, which confirms that the m_CSP may be at least partly made of collaterals of the pyramidal and cortico-striatal tracts^29^.

### Step 2: Optimization of tractography reconstruction parameters for accurate anatomical characterization of cortico-subcortical pathways in the NHP brain. The example of the motor cortico-subthalamic pathway (m_CSP)

**Figure 4:**
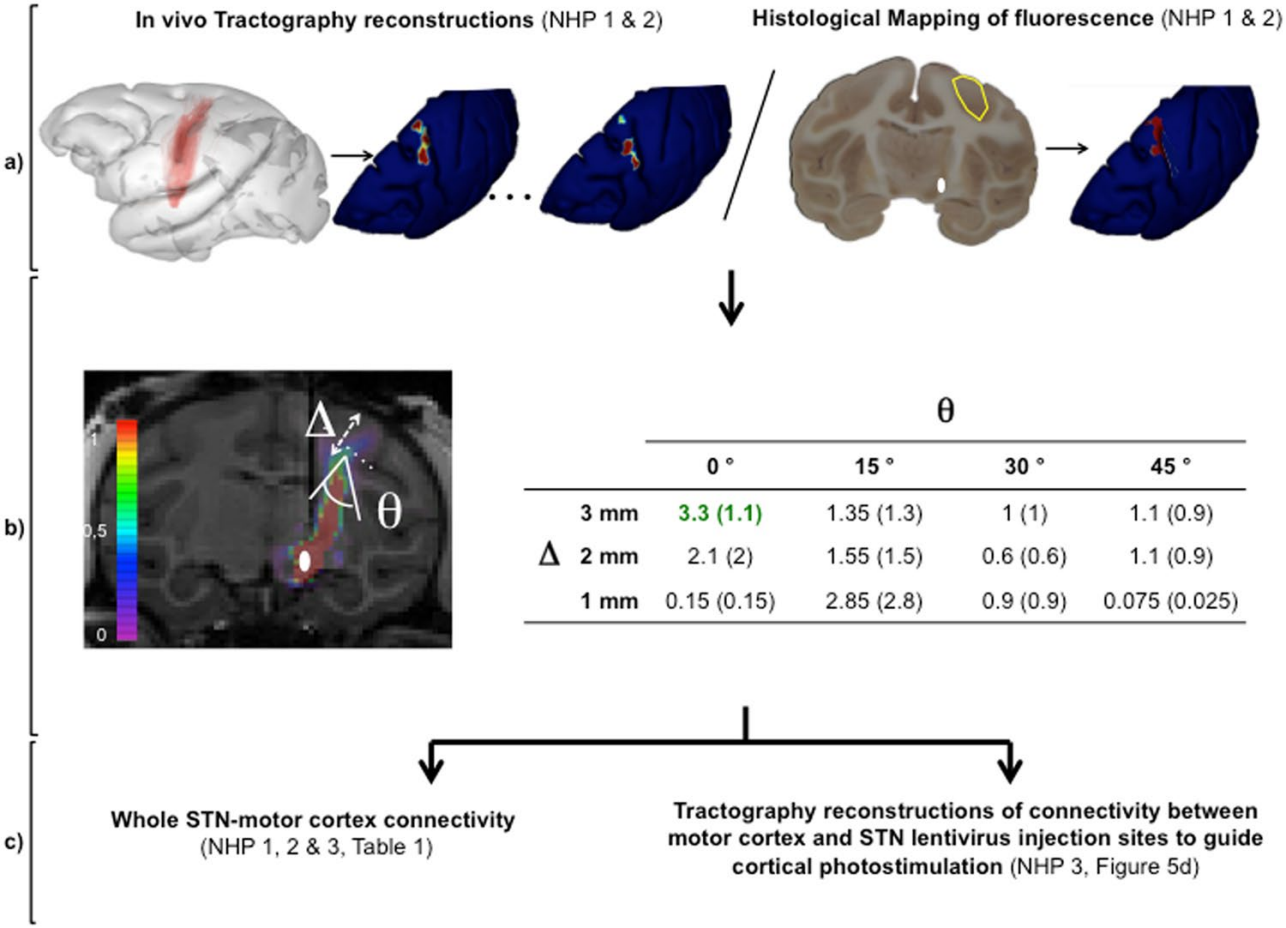
Histologically optimized Tractography in non-human primate. (**a**) In the same two non-human primates (NHP 1 & 2), *in vivo* tractography reconstructions after high angular resolution diffusion imaging (HARDI) acquisitions were performed for the pathway connecting the motor cortex to the lentiviral injection sites for various sets of reconstruction parameters. For these same animals, ChR2-eYFP associated cortical fluorescence was mapped in the motor cortex. Tractography and histological results were projected on cortical surface and experessed in a common space. (**b**) The optimal parameters (Δ, θ) for tractography reconstruction for each motor cortical area were those for which the average across NHP1 & 2 of the surface within the considered motor cortical area with local correlation was maximal (NHP 1 & 2). θ is the angulation between the normal to the cortical surface and the axis of fibers leaving the cortex. Δ is the depth from cortical surface for cortical origins of the studied fiber pathway. The probablity map of connection figure and the table illustrate the connectivity between primary motor cortex and the lentiviral injection sites in STN. The table displays values of the primary motor cortex surface (in mm^2^, (sem)) for which the correlation coefficient between tractography and histological cortical maps of connectivity is superior to 0.5, for various sets of reconstruction parameters. The values of Δ νδ θ for which the surface is maximal are considered to be the optimal reconstruction parameters between STN and the considered area of motor cortex, M1 in this case. Such parameters are determined for each considered cortical motor area and detailed in Table 1. (**c**) Determination of the optimal tractography reconstruction parameters between STN injection sites and motor cortical areas in NHP 1 & 2 thanks to comparison with histological findings enabled accurate determination of the connectivity between whole STN and motor cortical areas for both hemispheres of NHP 1, 2 & 3 on one hand, and to guide photo-detection and -stimulation of the primary motor cortex area with the highest density of connections with STN injection sites in NHP3 on the other hand. M1: primary motor cortex; m_CSP: motor corticosubthalamic pathway; NHP: non-human primate; sem: standard error of the mean; STN: subthalamic nucleus.

Anatomical characterization of m_CSP was performed by tractography reconstruction after *in vivo* High Angular Resolution Diffusion Weighted Imaging (NHP 1, 2 & 3). However, algorithms for tractography reconstruction of white matter tracts are highly sensitive to reconstruction parameters which have been empirically determined until now^30–32^. Reconstructions were based on parameters with direct anatomical relevance: cortical depth for the origins of m_CSP (Δ), and angulation of m_CSP with the normal to the cortical surface (θ) (Fig. 4b). Optimal parameters for each atlas functional area of interest were determined by comparison between tractography reconstructions and 3D-histological maps of ChR2-eYFP associated fluorescence in motor cortical regions, for NHP 1 & 2 (Fig. 4a and b). For this second step, the lentiviral vector with retrograde transport properties was used as a neuronal tracer.

These parameters enabled for the first time histologically guided reconstruction of m_CSP in NHPs (NHP 1, 2 & 3) and the performance *in vivo* of reconstructions of the white matter tract between lentiviral vector injection sites in the STN and primary motor cortex of NHP 3 in order to guide photostimulation of the primary motor cortical area with the highest density of connections to injection sites in the STN of NHP3 (Step 3). The whole procedure is detailed in Fig. 4.

#### Validation of the in vivo tractography reconstruction of the connectivity between motor cortex and injected sites within the STN of NHP 1 & 2 by comparison with post-mortem 3D-histological reconstructions

**Figure 5:**
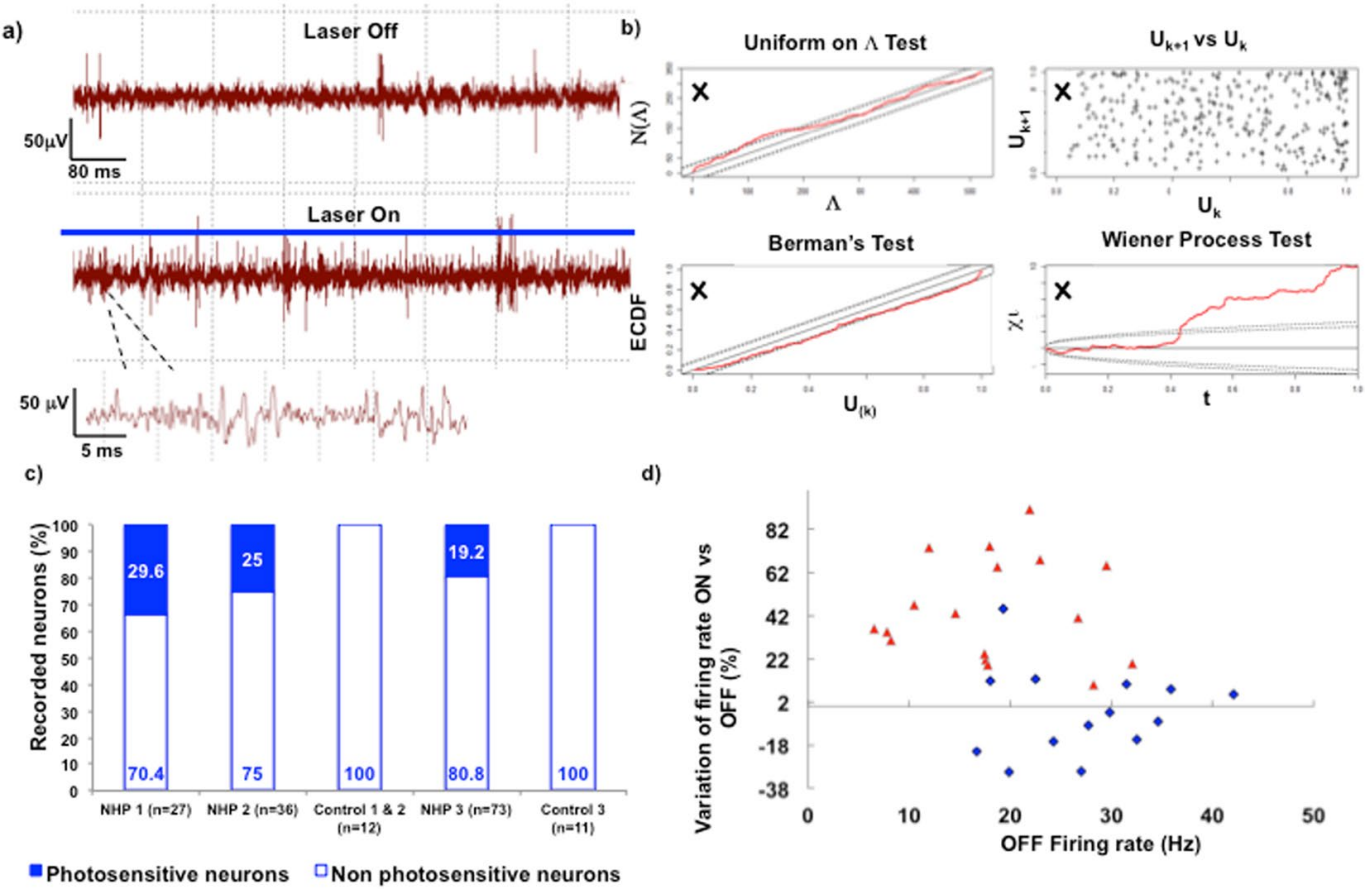
Electrophysiological validation of the lentiviral vector and functional characterization of the motor cortico-subthalamic tract in non-human primate. (**a**) Multi-unit recordings in the subthalamic nucleus without (upper trace) or with (lower trace) high frequency blue light photostimulation (NHP 2). (**b)** Identified after spike sorting by thresholding and principal component analysis, a unit was considered photosensitive by assessing the goodness of fit between the inhomogeneous poisson processes modelling the time series of its spikes occurrences during the OFF-prestimulation, ON-stimulation periods and post-stimulation periods (Supplementary Methods, section 6). Here the fit between poisson processes models for the OFF-prestimulation and the ON-stimulation periods of an STN unit is bad according to the four Ogata’s tests of goodness of fit. **(c)** The percentage of STN neurons that were photosensitive was determined during axon terminals (NHP 1 & 2) or m_CSP cortical origins (NHP 3) photostimulation. **(d)** Effects of cortico-subthalamic tract cortical origins (NHP 3, blue diamonds) or axon terminals (NHP 1 & 2, red triangles) photoexcitation on firing rate of STN photosensitive neurons. m_CSP: motor corticosubthalamic pathway; NHP: non-human primate; STN: subthalamic nucleus.

Spheres of 1 mm diameter centred on the sites of injection inside the STN were chosen as intersecting regions to select streamlines within tractograms reconstructed using tractography from high angular resolution diffusion weighted imaging (Fig. 5a and b). The cannulas visualized on the anatomical T1 MRI sequences helped identify the sites by measuring the distance between the tip of cannulas and the tip of the micro-needle at the sites of injection. Streamlines between motor cortical regions where fluorescence was detected (Fig. 3) and the injection sites within STN could be determined even though the m_CSP is much less dense than the pyramidal tract (Fig. 4a).

Local correlation projected on the motor cortical surface between the origins of the m_CSP defined by tractography and those of the 3D-histologically reconstructed CSP (H-CSP) were computed for each functional cortical area (see Fig. 4 a as an example for the connectivity between primary motor cortex and STN injection sites). The optimal parameters (Δ, θ) for tractography reconstruction for each motor cortical area were those for which the average across NHP1 & 2 of the surface within the considered motor cortical area with local correlation was maximal (Table 1, Supplementary information 10).

**Table 1:** Qualitative and quantitative anatomical description of the motor cortico-subthalamic pathway in non-human primates.

	Primary motor cortex	Premotor cortex	Supplementary motor area	Rostral cingular cortex	Caudo-dorsal cingular cortex	Caudo-ventral cingular cortex
**θ** (°)	0	45	45	45	30	0
**Δ** (μ)	3	2	2	3	2	3
% of all m_CSP fibers (sem)	49.2 (5.8)	23.6 (3.3)	19.9 (7.6)	7.3 (1.4)	2 (0.7)	0.4 (0.1)
% of cortical area surface projecting to STN (sem)	92.6 (3)	85.8 (2.5)	33.1 (8.7)	56.9 (17)	17.4 (6.1)	55.7 (16.9)

m_CSP: motor cortico-subthalamic pathway; sem: standard error of the mean; STN: subthalamic nucleus. (n = 6 hemipsheres).

#### Study of the connectivity between motor cortex and the whole STN using the parameters for tractography reconstructions validated by comparison with histological mapping of fluorescence

The whole STN was identified bilaterally on the histological blocks for NHP 1 & 2, and for NHP3 using a MRI sequence, developed for direct visualization of the STN^33^, whereas it had never previously been possible to precisely and reliably segment the STN on NHP MRI before. The primary motor cortex was found to be the main source of motor cortex projections to the STN (49.2 +/− 5.8% of all streamlines), followed by the premotor cortex (23.6 +/− 3.3%), supplementary area (19.9 +/− 7.6%), rostral cingular cortex (7.3 +/− 1.4%), caudo-dorsal cingular cortex (2 +/− 0.7%) and caudo-ventral cingular cortex (0.4 +/− 0.1%) (Table 1). Moreover the cortical origins of m_CSP were distributed over 92.6 +/− 3% of the primary motor cortex surface, 85.8 +/− 2.5% of the premotor cortex surface, 33.1 +/− 8.7% of the supplementary motor area surface, 56.9 +/− 17% of the rostral cingular cortex surface, 17.4 +/− 6.1% of the caudo-dorsal cingular cortex surface and 55.7 +/− 16.9% of the caudo-ventral cingular cortex surface (Table 1). We believe this is the first attempt to describe the motor cortico-subthalamic pathway in NHPs both qualitatively and quantitatively with the necessary precision for the efficient and safe cortical neuromodulation stimulation.

### Step 3: Functional characterization of the cortico-subthalamic pathway in non-human primate brain guided by tractography reconstructions

#### Transduction after retrograde transfer of ChR2-eYFP genes yielded a level of expression of ChR2 ionic channel on neuronal membranes high enough for photostimulation of the transduced neurons to be functionally efficient at both extremities of the motor CSP

The photostimulation parameters and the distance between the fibre tip and the recording electrode (cf Material & Methods, section 4; and Supplementary Figure 3) were chosen to avoid inducing non-specific neuronal responses possibly related to heating or photoelectric effects on the electrode. Such effects were never observed among the 12 control neurons for NHP 1 & 2, and the 11 control neurons for NHP3, and none of them was found to be photosensitive (Fig. 5c). The numbers of STN photosensitive neurons 2 months after viral vector injection were 8 out of 27 recorded neurons for NHP 1, 9 out of 36 recorded neurons for NHP 2. Fluorescence intensity at the level of injection sites within STN of NHP3 increased by 55.7% between D15 and D75, confirming that ChR2-eYFP transgenesis occurred (p-value = 0.002 Wilcoxon signed-rank test, Supplementary Figure 2b), leading us to try to perform cortical photostimulation guided by tractography reconstructions, while recording in the STN: 14 out of 73 recorded neurons for NHP 3 were found to be photosensitive (Figs 1, 5c and d, Supplementary Figures 4 and 5).

#### Stimulation of axon terminals and cortical origins of the CSP resulted in different effects on electrophysiological activity of STN neurons

When photostimulating at high frequency the axon terminals of the CSP in NHP 1 & 2, the 17 photosensitive neurons exhibited a significant increase of their firing rate by 44.9 +/− 5.5% (p < 0.0001, F(1.487, 23.79) = 23.05, ANOVA on repeated measures with Turkey’s multiple comparisons test after passed Shapiro-Wilk normality test) (see Fig. 5d, red triangles, for response of each neurone). The baseline firing rate was 18.2 +/− 1.9 Hz. During photostimulation, the average firing rate was 26.1 +/− 2.7 Hz (p < 0.0001 compared to baseline). During post-stimulation period, the firing rate was 17.8 +/− 1.7 Hz (p = 0.9 compared to baseline, and p = 0.0005 compared to photostimulation period).

Photoexcitation of m_CSP cortical origins elicited the emergence of distinct excitatory or inhibitory STN neuronal responses among photosensitive neurons (Fig. 5d). This was similar to the effect of electrical stimulation of the motor cortex that we previously reported in normal NHP too (Fig. 5c of ref.^27^).

## Discussion

The present study demonstrated that Optogenetic Tractography empowered anatomo-functional characterization of the motor cortico-subthalamic pathway in non-human primates with cell/network specificity and accuracy. Optogenetic Tractography, as demonstrated here, can be used to investigate any cortico-deep brain tract. This proof of concept study was performed in only a small number of normal NHPs for ethical reasons. The next step is to apply these technics directly to NHP models of neuropsychiatric diseases (^34^ for Parkinson’s disease;^35^, for Alzheimer’s disease:^36^, for depression;^37^, for addiction) with the aim of helping to refine targets for clinical neuromodulation by answering a number of key questions: Is the optimal target gray matter or afferent white matter for subcallosal cingulate gyrus CG25 DBS in depression^38^? Which tract, among the many tracts projecting to a given nucleus would be responsible for clinical benefit for DBS in addiction^39^? Which precise oscillatory bands should be used to modulate the various cortical origins of the motor cortico-subthalamic tract^40^? To develop in NHPs the technics described in this study we had to address several biotechnology challenges regarding vectorology, neuroimaging and neuromodulation.

The first step was to obtain precise, selective and robust long distance opsin gene transfer allowing high levels of safe expression across a specific cortical layer and over a large brain area from a connected deep brain hub in the NHP. In contrast to a direct cortical injection, this remote approach avoided any needle tract damage to the cortical tissue that could impair optical stimulation and analysis. Following the natural axonal pathway, led to opsins expression in a specific functional cortical area and at a specific layer of the entire connected gyrus, which would not be possible by direct cortical injections. This latter feature is of most importance, as red light can cross all the cortical layers and transcortical illumination may be safely used to neuromodulate a specific photosensitized layer^41^.

Thus, using a precise, safe and non-immunogenic long distance retrograde vector was a key feature of this optical approach. Here, we used EIAV lentiviral vectors pseudotyped with either Rabies glycoprotein or Rabies-G-VSVg-mainly because it allows robust retrograde gene transfer through binding to receptors located on axon terminals which increases targeting accuracy^42–44^. Thus, the retrograde gene transport obtained here in NHP 1 & 2 (up to 10,611 cortical labelled neurons) appeared to be effective in terms of number of transduced cells when compared to rodent experiments with ubiquitous promoters using the same lentiviral vector or to experiments with HIV-1 pseudotyped with Rabies glycoproteins in NHPs^21,44,45^. Given that retrograde serotypes of AAV vectors diffuse in larger volumes, and some even display trans-synaptic gene transfer^21^, we considered AAV vectors less appropriate to reach specific brain “hubs”.

Detailed *in vivo* 3D description of a cortico-deep brain tract is critical for any procedure aiming at stimulating a precise functional cortical area. To better characterize any tract, especially sparse ones like the m_CSP that sent collaterals to the pyramidal and cortico-striatal tracts, we implemented tractography reconstruction in NHPs with HARDI and not DTI. Indeed HARDI facilitates tracking of several pathways even when these are crossing in a given voxel^6,8^.

Tractography reconstructions after DTI or HARDI are both sensitive to the choice of anatomical parameters^30–32^ which have been chosen on an empirical basis up to now. Here, we demonstrated that parameters such as fibre angulation and cortical depth of penetration could be determined by comparison with 3D-histology reconstructions for each cortical area of interest, using the EIAV-Rabies-*ChR2* vector as a neural tracer.

Acknowledging that local diffusion of lentiviral vectors is limited compared with classical chemical tracers, we first determined these reconstruction parameters for the injection sites within STN which can be accurately identified on post-operative MRI and cortical fluorescence mapping in the same animals (NHP1 & 2). These parameters might vary depending on the location of injection sites within the STN. Additional animals and locations of injection sites within STN or any given deep brain nucleus should be included in further studies to refine the accuracy further. Nevertheless, we extrapolated these reconstruction parameters optimized for NHP 1 & 2 LV injection sites within STN, we reconstructed the whole NHP m_CSP (NHP 1, 2, 3) by applying these histologically-guided reconstruction parameters to the whole STN, and we performed efficient targeting of the m_CSP cortical origins for photo-stimulation with surgical accuracy (NHP 3). These technics will help in designing a photo-stimulation cortical device delivering light to a large brain volume matching closely the scattered cortical origins of the m_CSP: this will constitute a critical step to elicit behavioural changes in NHP models of neuropsychiatric disease. Our results show that the primary motor cortex is the principal source of afferents to the STN, followed by the premotor cortex and that their neurons projecting to STN are distributed almost on the entire surface. Previous trials of motor cortex electrical stimulation failed in PD patients^46^ but at that time tractography technics did not exist nor was the design of clinical cortical electrodes tailored to target an area as large as the primary motor cortex and premotor cortex.

Previous studies aiming at comparing histological identification of white matter tracts and diffusion MRI-based tractography in non-human primates of the pathways of interest^9–11^ were performed with chemical tracers. Chemical tracers may induce stronger retrograde labelling compared to LVs pseudotyped with Rabies glycoproteins. Indeed, retrograde ChR2-eYFP gene transport followed by transduction of remote connected neurons occurs after LV uptake from axon terminals of these remote neurons and depends on the density on axon terminals and its receptor density. But chemical tracers diffuse locally more than LVs, which is a problem when targeting a small deep brain nucleus surrounded with other nuclei. Moreover LVs equipped with ChR2-eYFP genes can enable, at the same time and in the same animal, both anatomical and spatio-temporal functional mapping at the scale of the neuron or even neuronal compartment and neuronal phenotype on one the hand, and at the milliseconds timescale on the other hand. Chemical tracers are not compatible with similar *in vivo* functional probing of neural pathways and cannot offer anatomical mapping potentially as refined as with LVs. These vectors enable spatial restriction/specificity by modifying the volume injected, titer, pseudotyping to target specific subpopulations of neurons or even subcellular compartments^47–49^, or to enable retrograde transport as preformed in our study, promoters to get tissue-specific gene expression (CaMKIIα for glutamatergic neurons^50,51^ or GAD67 for gabaergic neurons:^52^). LVs also enable temporal restriction/specificity by choosing: time of harvesting the tissue after the injection^53,54^, inducible promoters controlled by drugs or light^55,56^, post-translational regulation by light or drugs too^57^. Using promoters dependent on neural circuit activation such as the c-fos promoter, it is also now possible to label only neurons and pathways that were involved in a given task, and at a given moment^58^. Finally, with new tissue clearing techniques^59–61^ and microscope optics it is now possible to visualize transduced neurons in whole brains^62–64^, whereas chemical tracing requires slicing the brain and manually recording the axonal termini. Here we exploited specific LV pseudotypes, type of tissue specific promoter and combination with tractography technics to demonstrate how LVs combined with optogenetics and appropriate imaging technics can be used to efficiently and accurately allow anatomo-functional mapping in NHPs. This technology can be refined and combined with other imaging/clearing technics for additional insights into the neuronal function and neuronal circuitry.

Electrophysiological studies were carried out in a small number of healthy animals as a proof of concept and for ethical reasons. Drawing conclusions regarding basal ganglia neurophysiology will require a larger number of healthy and disease model animals. Nevertheless, photo-stimulation of both extremities of the m_CSP modified electrophysiological activity of STN neurons, validating the efficacy of ChR2 transgenesis along a cortico-subcortical pathway in NHPs after retrograde gene transfer with an EIAV-Rabies +/− VSVg vector. Photo-excitation of cortical origins and axon terminals of the m_CSP produced different electrophysiological responses among STN neurons (Fig. 5d). So even if photo-stimulation and electrical stimulation of both extremities of the m_CSP were reported to produce similar motor improvements in 6-OHDA rodents^15^ and MPTP monkeys^2,27^, the mechanism of action may not be the same. The inability of wild-type ChR2 to respond to high frequencies^13^ and differences in expression density on somatic or axonal membranes may have accounted partially for the disparities observed in our study between cortical and axon terminals electrophysiological responses. A further exploration of the differences between cortical versus axon terminal stimulation, in MPTP NHPs using optogenetic tractography may help elucidate why previous clinical trials of cortical electrical stimulation failed to correct motor symptoms in PD patients and perhaps how to optimize the procedure to achieve an effective therapy^46^. Cortical neuromodulation would be an interesting strategy for functional neurosurgery since it enables selective and remote stimulation of a targeted functional area of a deep brain nucleus to reduce side effects^27,65^, while being a less invasive and faster procedure from a neurosurgical point of view.

Cetin *et al*.^45^ used in mice a similar EIAV lentivirus pseudotyped with Rabies glycoproteins: after injecting a similar number of vector particles as reported in this study, taking into account vector concentration and injected volumes, they could not elicit photocurrents nor synaptic currents at the injection site. Other differences include the following; they could not directly identify and record from transduced neurons, the analysis was at three weeks post injection and not two months, the investigated brain structures, the species, the preparation, and the electrophysiological analyses. Moreover in NHP1 & 2 we photostimulated above the dorsolateral aspect of the STN, where axons of the m_CSP converge towards the STN, so that a sufficient number of these axons may have been recruited to elicit modifications of spikes distribution in 25–29.6% of recorded STN neurons in NHP 1 & 2. For NHP 3, functionally successful stimulation of photosensitive neurons scattered in the motor cortex may have benefited from the use of the chimeric pseudotyping of the EIAV lentiviral vector (pSA91CVSVSVG) which has been shown to enable stronger retrograde transport^44^ and from the guidance of *in vivo* histologically-optimized tractography reconstructions to accurately identify the cortical area with the highest probability of being photosensitive. Future studies should aim at increasing retrograde transfer efficiency of pseudotyped lentivirus for broader expression of red-shifted opsins with higher kinetics, while avoiding potential toxicity^41,66^.

The translational potential of Optogenetic Tractography relies on deciphering normal and pathological neural networks involving cortical and deep brain structures in NHP models of neuropsychiatric diseases. From a functional point of view, the goal of our method is to identify therapeutic neuromodulation targets that could be translated to patients with neuropsychiatric disorders. From an anatomical point of view, due to the fact that human brain has different patterns of sulcus folding from macaque monkeys, the tractography reconstruction parameters we identified in this study might not be directly translated to patients. But in humans, it might be possible to perform similar investigations post mortem with injections of chemical tracers^67^ in combination with tractography. Above all we imagine that the uncertainty of tractography reconstructions of sparse cortico-subcortical tracts using HARDI might be compensated to some extent by the new generation of directional DBS leads enabling steering of the electrical field^68^.

## Methods

### Animals and Housing

All animal studies were conducted according European (EU Directive 86/609) and French regulations (French Act Rural Code R 214-87 to 131) and the experimental protocol (n°12–077) was approved by the local ethics committee of the french Alternative Energies and Atomic Energy Commission (CEA): Committee of Ethics in animal experimentation number 44. The animal facility is authorized by local veterinarian authorities (authorization n° A 92-032-02) and complies with Standards for Humane Care and Use of Laboratory Animals of the Office of Laboratory Animal Welfare (OLAW – nu#A5826-01). All efforts were made to minimize animal suffering and animal care was supervised by veterinarians and animal technicians skilled in the healthcare and housing of NHPs. All animals were individually housed under standard environmental conditions (12-hour light-dark cycle, temperature: 2261uC and humidity: 50%) with free access to food and water. Experiments were conducted on a total of three male rhesus monkeys (Macaca mulatta, supplied by Noveprim, Mauritius Island) of a mean age of 5 years and a mean weight of 6.5 kg.

### Anatomical Magnetic Resonance Imaging

Animals were anesthetized with 10:0.5 mg/kg ketamine/xylazine and placed in the magnet in a sphinx position, fixed by mouth and ear bars to a stereotactic MRI-compatible frame (M2E, France). Once in the magnet, animals were heated by a hot air flux and their temperature and respiration parameters monitored remotely.

For surgical targeting of the subthalamic nucleus, MRI was performed on a 7 Tesla horizontal system (Varian-Agilent Technologies, USA) equipped with a gradient coil reaching 100 mT/m (300 ms rise time) and a circular radiofrequency 1 H coil (12 cm inner diameter). T2-WI were acquired using a fast spin-echo sequence with the following parameters: TR = 4750 ms, effective TE = 62 ms, acquisition time = 16 min, FOV = 115 × 115 mm and matrix = 256 × 256 resulting in a 450 × 450 µm in plane resolution, 40 coronal slices, slice thickness = 1 mm.

For tractography reconstruction of the motor cortico-subthalamic pathway, an imaging protocol was performed on a 3 T Tim Trio MRI system (Siemens Healthcare, Erlangen) equipped with a whole body gradient system (Gmax = 40 mT/m, slewrate of 175 T/m/s) and using birdcage coil including a sub-millimeter T1-weighted MPRAGE imaging scan, a 2D single shot twice-refocused spin echo EPI high angular resolution diffusion imaging (HARDI) scan, a 3D T2 FLAIR imaging scan and a fieldmap calibration scan.

Detailed parameters of the MPRAGE T1-weighted sequence: TE/TR/TI = 3.18 ms/2000 ms/900 ms; FOV = 154 mm; 4 averages; matrix 192 × 192; flip angle 9°; 144 slices thickness TH = 0.8 mm leading to an isotropic resolution of 0.8 mm; scan duration 28 min11s

Detailed parameters of the HARDI sequence: TE/TR = 90 ms/6000 ms; FOV = 120 mm; matrix 72 × 72; 30 slices; slice thickness TH = 1.7 mm yielding an insotropic voxel resolution of 1.7 mm; read bandwidth = 1694 Hz/pixel; partial Fourier factor 5/8; no parallel acceleration factor; no multiband acceleration; 256 diffusion directions uniformly distributed over the unit sphere plus 2 null b-value; b-value of 1500 s/mm2; 2 repetitions; scan duration of 51min36s.

Detailed parameters of the 3D T2-weighted FLAIR sequence: TE/TR/TI = 649 ms/6000 ms/2000 ms; FOV = 192 mm; slice thickness TH = 0.75 mm; 4 averages; matrix 256 × 256; phase FOV = 75%; partial Fourier factor 7/8; turbo factor 191; echo spacing 3.74 ms; echotrain length 1358; read bandwidth = 698 Hz/pixel

Detailed parameters of the 2D B0 fieldmap Gradient Echo calibration: TE1/TE2/TR = 4.92 ms/7.38 ms/500 ms; FOV = 146 mm; matrix 86 × 86: 30 slices; slice thickness TH = 2 mm; flip angle = 60°; read bandwidth = 328 Hz/pixel.

### Lentiviral vector construction and production

The pONY series of EIAV vectors and their pseudotyping with the different envelopes have been described previously^69,70^. The rabies glycoprotein (pHGKCVS) used to pseudotype the EIAV-Rabies-CaMK2-*ChR2-eYFP* vector was from the Challenge virus standard (CVS) strain. For the EIAV-Rabies-VSVg-CaMK2-*ChR2-eYFP* vector a chimeric glycoprotein (pSA91CVSVSVG) composed of the N terminal extracellular and transmembrane domains of the CVS rabies glycoprotein and the C-terminal cytoplasmic domain of the VSV glycoprotein was constructed based on data published by Kato *et al*.^44^.

The EIAV-Rabies-CaMK2-*ChR2-eYFP* vector stock was generated by FuGENE 6 (Roche, UK) transfection of human kidney 293 T (HEK293T) cells plated on ninety 10 cm dishes (3.5 * 10^6^ cells/dish) with 4 µg of vector plasmid (pONY8.9camIIK*ChR2-YFP*), 2 µg of gag/pol plasmid (pESGPK) and 0.3 µg of rabies-G (pHGKCVS) plasmid per dish, according to the manufacturer’s instructions. The EIAV-Rabies-VSVg-CaMK2-*ChR2-eYFP* vector was produced by Lipofectamine™ 2000 CD (Invitrogen, Cat. 12566-101) transfection of HEK293T cells (3.5 * 10^6^ cells/dish) plated on one-hundred and thirty 10 cm dishes with 4 µg of vector plasmid (pONY8.9camIIK*ChR2-YFP*), 2 µg of gag/pol plasmid (pESGPK) and 0.6 µg of CVS/VSV-G chimeric glycoprotein (pSA91CVSVSVG) plasmids per dish, according to the manufacturer’s instructions. 14–18 h after transfection, sodium butyrate was added to a final concentration of 10 mM. Media was changed 6–8 h after sodium butyrate induction, and 21–23 h later vector was harvested and filtered through a 0.45 µm syringe filter and the vector concentrated 2000-fold by centrifugation. This comprised an initial low speed centrifugation at 6000 g at 4 °C for a minimum of 18 h, followed by ultracentrifugation at 50,000 g at 4 °C for 90 min. The final vector products were re-suspended in TSSM formulation (Tromethamine, NaCl, Sucrose and Mannitol). The vector titres, in transducing units/ml (TU/ml), were estimated by integration (DNA) titre assay^71^ and the titre of EIAV-Rabies-CaMK2-*ChR2-eYFP* was 8.1 × 10^7^ TU/ml, and from EIAV-Rabies-VSVg-CaMK2-*ChR2-eYFP* was 5.2 × 10^7^ TU/ml.

### In-depth *in vivo* measurement of fluorescence and photostimulation with a home-made minicube

To validate the expression of *ChR2-eYFP* in the STN, we measured the amount of tissue fluorescence following the injection of our lentiviral vector. Fluorescence measurements were performed through a single bare optical fibre (probe fibre, 400 µm, 0.48 NA) acutely implanted into the brain and connected to a custom fluorescence detection cube (FDC). To ensure proper light delivery and collection at the implanted fibre tip, a flat endface was obtained by cleaving the probe fibre. The FDC was built as a regular filter cube with a bandpass excitation filter (FF01-480/40, Semrock), a dichroic beamsplitter (FF518-DiO1, Semrock), a longpass emission filter (LP02-514-RU, Semrock) and a set of aspheric lenses. In addition, it contained a lens after the emission filter used to form an image of the probe fibre onto the detector of a CMOS board camera (DMM 72BUC02-ML, The Imaging Source). The board camera was connected to a computer through a USB link and acquired images were analyzed with ImageJ (Fig. 3).

Two excitation light paths (one from a cyan LED, 505 nm, Doric Lenses, and one from a 473 nm DPSS laser, Shangai Laser) were combined into the source fibre via a filter cube (beam combiner), allowing fluorescence measurement (LED ON, laser OFF, *typically 21* microWatt, i.e. 0.67 mW/mm^2^ at the probe fibre tip, 0.8 s exposure times) and *ChR2* excitation (LED OFF, laser ON) through the same probe fibre. The beam combiner contained a bandpass excitation filter for each light source, a dichroic mirror (LM01-480, Semrock) and a set of aspheric lenses for beam collimation and focusing. The laser was controlled via TTL pulses and 5 ms long pulses were delivered at high frequency (130 Hz) for 30 to 90 seconds during electrophysiological experiments. Knob on the laser was adjusted so that power irradiance at the tip of the fibre optics measured before each surgery with a powermeter (Thorlabs PMD) was 200 mW/mm^2^. Deep brain photostimulation was performed with the fibre located in one of the two channels of the cannula, typically one mm above the dorsal-lateral aspect of STN, and with the STN recording electrode within the second channel of the cannula, for two reasons: 1/ avoid direct photostimulation of recorded neurons and non-specific neuronal responses possibly related to heating or photoelectric effects on the electrode; 2/ try to stimulate a large portion of the photosensitive m_CSP in order to increase the probability of recording STN neurons receiving photosensitive and photostimulated m_CSP afferents.

Image analysis consisted in measuring the average fluorescence intensity (average pixel intensity) with ImageJ software. Ten measures of fluorescence inside STN of the injected hemisphere, along the canula main axis, with 100 microns space between each imaging site, and centered on gravity centre of injection sites, were performed in the STN at D15 and D75 for NHP3, and were compared using a Wilcoxon signed-rank test.

## Electronic supplementary material

Supplementary Information
