# Supplementary material for: Optogenetic Tractography for anatomo-functional characterization of cortico-subcortical neural circuits in non-human primates
Source: Sci Rep. 2018 Feb 20;8:3362. doi: 10.1038/s41598-018-21486-8 (PMC5820256; doi:10.1038/s41598-018-21486-8)
Supplement: Supplementary file 1 — Supplementary Information [file 41598_2018_21486_MOESM1_ESM.doc]

**Optogenetic Tractography**

**for anatomo-functional characterization of cortico-subcortical neural circuits in non-human primates**

S. Senova, C. Poupon, J. Dauguet, H. J. Stewart, G. Dugué, C. Jan, K. Hosomi, G. S. Ralph, L. Barnes, X. Drouot, C. Pouzat, J.F. Mangin, F Pain, I. Doignon, R. Aron Badin, E. Brouillet, E. Boyden, K. A. Mitrophanous, P. Hantraye, and S. Palfi

**SUPPLEMENTARY FIGURES**

**Supplementary Figure 1: Histological evaluation of blue light high frequency longlasting photostimulation effect on neuronal density and activation in a rat.**

Photostimulation was performed in a rat brain with same fiber optics (400 μm, 0.48 NA) and stimulation parameters: 130 Hz, 200mW/mm^2^, 5 ms pulsewidth, 90s long as experiments in non human primates. Fifteen cortical cryosections (10µm) were labeled for Neu-N (red) (a & c) and c-fos (green) (b & d) and. Cortical Neurons and activated neurons were quantified for photostimulated side (left column) versus control one (right column) on respectively Neu-N stained and c-fos stained slices centered on the spot of maximal light intensity. Scale bar = 40µm. Toxicity related to high frequency long-lasting blue photostimulation in the absence of vector injection was evaluated in a rat for ethical reasons. There was no decrease of neuronal density in photostimulated areas (NeuN counting: 470 +/- 18.3 neurons/ROI on control side vs 482.1 +/- 12.6 neurons/ROI on photostimulated side, p= 0.29, Mann Whitney test), nor histological sign of neuronal activation (cFos counting: 15.4 +/- 2.74 neurons/ROI on control side vs 14.7 +/- 1.79 neurons/ROI on photostimulated side, p= 0.41, Mann Whitney test).

**Supplementary Figure 2: Deep brain and in vivo fluorescence detection and photostimulation in the non human primate.**


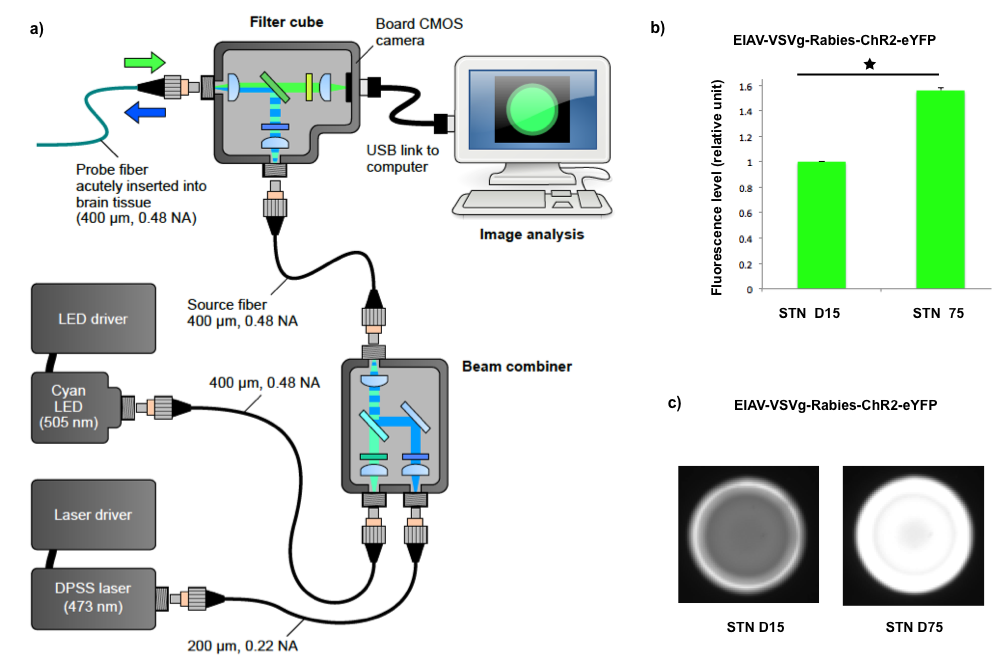


**a)** description of the device enabling consecutive deep brain and in vivo fluorescence detection and cerebral photostimulation in the non human primate with the same fibre optics ending in the primate brain.

**b)** At D15, day of the injection of EIAV-VSVg-Rabies-ChR2-eYFP vector in STN, and then at D75, fluorescence was detected in the STN of NHP3. Fluorescence increased STN by 55.7% between D15 and D75 (n=10 imaging sites, p-value=0.002 Wilcoxon signed-rank test).

**c)** Image recorded by the device in the STN of NHP3 at D15 and D75.

Fibre output power = 21 microWatts; time of exposiiton= 0.8 s.

**Supplementary Figure 3: Stimulation and recording configuration for NHP 1 & 2**

**Supplementary Figure 4: Stimulation and recording configuration for NHP 3**

**Supplementary Methods**

1. **Neurosurgeries (Figure 1).**

At D0, MRI-compatible recording chambers (Crist Instruments) were chronically implanted, centered on the STN targeted by direct visualization on T2-weighted images (T2-WI).

At D7, MRI-compatible canulas (internal diameter= 650 microns) for vector injections and STN electrophysiological recordings were chronically implanted inside the chamber. Distal extremity was 1 mm above the dorsal border of the dorso-lateral region of the STN. Targeting of the STN was again made by direct visualization of STN on pre-operative T2-WI, but was also confirmed both by recording of the well-known typical electrophysiological activity of STN, and per-operative T2WI before anchoring the canulas with dental cement.

At D15, viral vector injections were performed.

At D15 (as control experiments for photostimulation (NHP1 & 2) and fluorescence (NHP 3), just before viral vector injection) and at D75, in-depth *in vivo* measurement of STN fluorescence (NHP 3), photostimulation and electrophysiological recordings within STN were performed through the canula (NHP 1 & 2) and/or through a burrhole performed above motor cortex identified as projecting to STN according to DWI and tractography reconstructions (NHP 3). Time interval between sessions of photostimulation and electrophysiological recordings was typically one week.

1. **Viral vector injection.**

NHP were anesthetized with 10:0.5 mg/kg ketamine/xylazine (IM) and placed in a MRI-compatible stereotactic frame in a sphinx position, fixed by mouth and ear bars. The vectors described above were injected in dorsolateral part of the STN through a 50μl Hamilton syringe connected to an injection micropump (KDS, France) at a rate of 1 μl/min. Pre-op and perioperative 7T MRI enabled direct visualization of the STN and were challenged by perioperative electrophysiological targeting of the STN. NHP 1 & 2 were injected within STN with the EIAV-Rabies-CaMk2-*ChR2-eYFP* as described in Figure 1-b: 2,5 μl per site, in 5 sites for NHP1; and 10 μl per site in 2 sites for NHP2 . For NHP 3, we performed two injections distant of 1 mm, of 10 μl of EIAV-Rabies-VSVg-CaMK2-*ChR2-eYFP* along an orthogonal trajectory in a coronal plane. We chose to inject such large volumes guided by the studies published by Kato et al (1), who injected 7.5 μl of a Lentivirus-Rabies-VSVg-GFP per site in non human primates, and by Celtin et al (2) who injected 0.5 μl of EIAV-Rabies-ChR2-YFP (2.8*10^9^ TU/ml) per site whereas our viral vector had a titer of 8.1 x 10^7^ TU/ml. We want to highlight that in a phase ½ clinical trial, we used safely EIAV-lentiviral vectors for restorative dopaminergic gene therapy, without pseudotyping with Rabies glycoproteins, and with titers of 1.9 x 10^7^ TU/ml, 4.0 x 10^7^ TU/ml and 1 x 10^8^ TU/ml, showing a dose-response effect (3). Such titers were chosen as a compromise between efficacy and safety, especially for long term experiments as with NHP.

1. **Recordings.**

Multi-unit electrophysiological activity was recorded using microelectrodes (microTargeting^TM^ electrode, FHC, Bowdoin, ME; impedance 0.8-1.5 MΩ at 1 kHz) at D15 (control experiments) and from D75. Electrophysiological activity was amplified and recorded through a 500 to 3000 Hz band pass, using a Leadpoint^TM^ system (Medtronic, Minneapolis, USA). The penetration of the cannula was stopped at the entrance of the STN (Fig. 1 A).

At each recording site within the STN, recordings included first a baseline of at least 60 seconds, the OFF-prestimulation period, then an ON-photostimulation recording period of 30 to 90 seconds starting typically 10 s after beginning of the photostimulation to avoid network transitory effects (4) and finally a recording period of 60 to 90 seconds, the OFF-poststimulation period. Such long periods of recordings per recording site were chosen because of the known latency up to tens of seconds for clinical benefits of STN DBS to appear or to disappear in PD patients (5). But because of the timelentgh of each trial, it was not possible to repeat several trials per recording site, because neurons could not be stably recorded for more than 10 minutes typically. That is why we chose a procedure to determine neuronal photosensitivity which requires spike trains analyses for one trial per recording site only (cf paragraph 5 of Supplementary information). For NHP 1 & 2, axon terminals of the cortico-subthalamic (CSP) pathway were photostimulated whereas for NHP 3, cortical origins of the CSP were photostimulated in primary motor cortex as guided by *in vivo* tractography reconstructions after DWI.

For NHP 1 & 2, control experiments were performed just before the injection of viral vector, the day of the injection, with the same parameters of photostimulation as used at D75: none of the STN recorded neurons satisfied the photosentivity criterion when axon terminals of the CSP were photostimulated (200mW/mm^2^, 5ms pulsewidth, 130 Hz) (Figure 5 c). For NHP 3, cortical controls were performed by photostimulating M1 and recording in the STN in the controlateral non-injected hemisphere at D75 with the same stimulation parameters.

1. **Spike Sorting.**

Single-unit activity (SUA) was determined after spikes detection by thresholding taking into account the signal standard deviation and template matching, controlled by cluster analysis with principal component analysis and final visual inspection using DATAview software. The frequency of isolated SUA of STN had to be between 5 and 40 Hz before photostimulation to be considered for analysis. Furthermore, only SUA that were stable during the 60 seconds baseline were kept for further analysis.

1. **Spike trains analysis.**

The spike trains of SUA for OFF-prestimulation, ON-stimulation and OFF-poststimulation were analysed for modelisation with an inhomogeneous Poisson process and then goodness of fits were compared between spike trains resorting to the four Ogata’s test simultaneously, using the software STAR (Statistical analysis with R). A neuron was considered photosensitive if the model fitting the OFF-prestimulation spike train was not fitting at least 30 seconds of the ON-stimulation spike train according to at least three of the four Ogata’s statistical tests, and according to at least one more test than for the comparison between the OFF-prestimulation spike train and at least 30 seconds of the OFF-poststimulation spike train .

1. **Euthanasia and Tissue Preparation.**

Animals were anesthetized with 10:0.5 mg/kg ketamine:xylazine. A lethal dose of pentobarbital was delivered before transcardial perfusion with cold 0.9% NaCl. Brains were extracted and immediately placed in ice-cold 4% paraformaldehyde for 5 days and sucrose-containing phosphate buffer gradients for cryoprotection before immunohistochemistry.

1. **Immunohistochemistry.**

Whole brains were sliced into 40 microns-thick sections using a freezing microtome. They were washed and permeabilized 3 times for 30 minutes each in a solution containing PBS, 100 mM glycine, and 0.5% triton X-100 (PTG solution), and then blocked for 2 hours in PTG + 2% normal goat serum from Jackson Immunochemicals (PTB solution). Slices were then incubated with primary antibody in PTB solution overnight at 4°C on a shaker, then washed 4 times for 30 minutes each with PTG solution, and then incubated in the secondary antibody in PTB solution for 2 hours at 11 room temperature. Finally, the slices were washed 3 times for 30 minutes each with PTG solution, and for 30 minutes in PBS + 100 mM glycine. Antibodies and stains used were rabbit anti-alpha-CaMKII (1:50, Santa Cruz Biotechnology), rabbit anti-GFAP (1:1000,Upstate), mouse anti-NeuN (1:1000, Chemicon), chicken anti-eYFP (1:500, Chemicon), guinea pig anti-VGluT1 (1 :2500), Alexa 488 goat anti-chicken (1:500), and Alexa 568 goat anti-rabbit (1:500) (Invitrogen). Slices were mounted with Vectashield solution (Vector Labs), and visualized with a Zeiss LSM Pascal confocal microscope.

Confocal data analysis of immunostaining and cell counting was performed by taking 3-D z-stacks with a 63x oil lens (Zeiss), each 202 x 202 x 60 microns in volume. To

optimize accuracy of overlap determination and cell counting, cells were first identified on the green (*ChR2-GFP*) and red (NeuN) channels separately, then overlap was assessed.

1. **3D-Histology.**

**a- Mapping fluorescence into MRI space.**

For mapping fluorescence detected on histological slices with *in vivo* 3D MRI scans, the following procedure was followed. First, regions of fluorescence composed of soma, dendrites and axons were manually segmented on histological slices using an Olympus fluorescence microscope with a X40 objective with Mercator software (Explora Nova, La Rochelle, France). Approximately 12 sections per macaque with an inter slice spacing of 640 µm were showing fluorescence in the motor cortex region. External contours of the slices were also traced to provide a macroscopic reference landmark. Each histological slice was also digitized using a flat bed ImageScanner III (General Electric Heathcare LifeSciences) in transmission mode at a macroscopic resolution of 800dpi. Using the contours previously delineated on the microscope, segmented fluorescence areas were mapped onto the corresponding digitized histological slices using a rigid transformation estimated manually with BrainVisa toolbox. During cutting of the brain, blockface photographs were taken repeatedly at the exact same position spanning the whole brain. The direct stacking of the photographs provided a photographic volume restoring the spatial consistency lost on individual 2D histological slices. Each digitized histological slice was registered with the corresponding photograph using a composition of 2D rigid and affine transformations estimated using a robust blockmatching strategy with correlation coefficient as similarity criterion followed by a cubic B-spline transformation with mutual information similarity measure as described in (6) implemented in the Insight Tool Kit (KitWare). For mounted histological slices presenting tears, folding or missing parts, an extra thin-plate splines transformation was estimated by manually clicking corresponding anatomical points both on the photograph and the digitized slice (Insight ToolKit). The photograph volume was finally registered with the *in vivo* T1 MRI using a composition of 3D rigid, affine transformations and 3D cubic B-spline transformation. The complete series of transformations were composed to map in 3D the regions of fluorescence into the MRI space.

**b- 3D Digital Atlas warping.**

We used the MNI macaque atlas (7) which is composed of a template MRI and a corresponding 3D label image. The template MRI is based on the average of 25 normal adult macaque monkeys (18 Macaca fascicularis, 7 Macaca mulatta). The label image is the corresponding Paxinos segmentation of the template brain. We first checked the label image and cleaned out small artefacts of the atlas by removing non-consistent small connected components for each label. The provided atlas represents only one hemisphere so we operated a symmetrization of the atlas label image by computing the mid-sagittal plane of the template MRI (8) and then applied a symmetry along this plane of the label image. This resulted in a whole brain label image that we used as the atlas label image in the following.

For each non human primate in this study, we estimated a non rigid transformation mapping the template MRI of the atlas to the MRI of each animal using a composition of 3D rigid, affine transformations and 3D cubic B-spline transformation.

We then applied the transformation estimated for each animal to the atlas label image so that the Paxinos labels were mapped onto the individual MRI of each animal. The resulting warped atlas for each animal was used in the following to compute brain region statistics.

1. **DWI and Tractography reconstruction.**

The analysis of the T1-weighted MRI data acquired at 3T was performed using BrainVISA/Morphologist and included a correction of the intensity inhomogeneity, the extraction of the brain mask from which the pial surface of the cortex was extracted. A manual segmentation of the basal ganglia was also performed from the T1-weighted MRI data. A further parcelation of the cortical mantel was obtained from Paxinos atlas to study the connectivity of the deep brain structures based on Brodmann's functional areas.

The inference of the connectivity from the HARDI dataset was performed using Connectomist (9) and consisted of 1) correcting all the imaging artifacts (correction of Chi non-centered noise, spike detection and correction, removal of the geometrical distortions induced by eddy currents including the correction of the corresponding diffusion directions, correction of the distortions due to susceptibility effects using the B0 fieldmap calibration), 2) determining the affine transformation to match the Diffuson Weighted dataset to the anatomical T1-weighted and FLAIR datasets, thus insuring a perfect match between them, 3) computing a field of local HARDI model (analytical Q-ball model with spherical harmonics order 6 and regularization factor 0.006 (10) on the entire brain, 4) computing a robust mask of the brain using the T1-weighted dataset, thus defining the domain of propagation for the tractography algorithm to avoid the use of FA-based masks too sensitive to the choice of the FA threshold, and 5) computing the whole brain connectogram using two different streamlining fiber tracking algorithms (deterministic and probabilistic), using 8 seeds per voxel of the propagation domain, and setting the following streamlining parameters: forward step of 0.4mm and maximum aperture angle of 30 degrees, yielding millions of numerical tracts (11).

Based on the knowledge of the tractogram, of the cortical surface and of the segmented deep structures including the STN, a connectivity matrix was computed between all the structures and the cortex for each tracking method. A similar connectivity matrix was also computed between the structures and the functional cortical areas of interest defined using the Paxinos atlas, and the connectivity profile of STN was projected onto the cortical surface individually, using a dedicated connectomics pipeline. End-points for connection of a streamline to the cortex were determined by the distance between a “connecting” point belonging to a streamline and the cortical surface, and by the angle between the tangent to the streamline at the “connecting” point and the normal to the cortical surface, which must remain below a given threshold to avoid the creation of artifactual connections when a streamline moves tangentially to the cortex but does not connect to it. Four angular thresholds were tested: 15°, 30°, 45°, 90°.

1. **Correlation between 3D-Histology and Tractography reconstruction.**

Since the regions of fluorescence were mapped to 3D data and matched to the anatomical T1-weighted MRI, it was possible to integrate the information of fluorescence along the normal direction to the cortical surface and to project the information onto the pial surface. In order to be able to map the information at different depth levels, the integration of the signal along the normal of each vertex of the surface was performed with the following configurations of the integration boundaries 0mm-2mm, 0mm-3mm. The functional areas stemming from the Paxinos atlas matched to the individual T1-weighted MRI were also intersected with the pial to define functional areas of interest. Then, a correlation analysis performed between the patch of connectivity of the injection site onto the cortical surface computed obtained using diffusion-based tractography and the projection maps of fluorescence, yielded a map of correlation coefficients for all the individual functional areas, for the various depth levels and angular thresholds. Last, for each functional area, the proportion of the area depicting a correlation greater than a given threshold was determined for the following correlation thresholds: 0.1, 0.2, 0.3, 0.4 and 0.5, for both probabilistic and deterministic algorithms. For a given motor cortical area, the reconstruction parameters for which area of correlation >0.5 was maximal were chosen. If for all the investigated reconstruction parameters, the area for which correlation >0.5 was <2mm^2^, the other thresholds of correlation were taken into account to determine the optimal reconstruction parameters. The chosen parameters were those maximizing the area of correlation between histology and tractography averaged across the two animals and the two reconstruction algorithms.

1. **Safety of long-lasting high frequency blue photostimulation on brain tissue: assessments of cellular activation and neural density (Supplementary Figure 1).**

Experiments were performed on a 300g rat that was anesthetised with i.p. injection (0.3ml/ 50g) of a solution made with 20% ketamine, 8% dormitor, 72% distillated water. A temporal craniotomy was performed. The extremity of a 400 microns fibre-coupled LED was applied on the lateral portion of the brain and 130Hz, 5ms pulsewidth, 200mW/mm^2, 473nmm photostimulation was performed for 90s. Then histological staining and quantifications were performed on the sliced brains with c-fos and NeuN antibodies comparing the photostimulated hemisphere and the non-photostimulated hemisphere. Regions with same surface were chosen across 15 consecutive brain sections centered on the location of fibre optics. For both hemispheres, positive Neu-N neurons and positive c-fos cells were counted: values for photostimulated and non-photostimulated hemispheres were compared with a Mann-Whitney test.

**REFERENCES OF SUPPLEMENTARY METHODS**

1. Kato, S. et al. A lentiviral strategy for highly efficient retrograde gene transfer by pseudotyping with fusion envelope glycoprotein. Hum Gene Ther. 2011 Feb;22(2):197-206.
2. Cetin, A., Callaway, E.M. Optical control of retrogradely infected neurons using drug-regulated "TLoop" lentiviral vectors. J Neurophysiol. 2014 May;111(10):2150-9.
3. Palfi, S. et al. Long-term safety and tolerability of ProSavin, a lentiviral vector-based gene therapy for Parkinson's disease: a dose escalation, open-label, phase 1/2 trial. Lancet. 2014 Mar 29;383(9923):1138-46.
4. Gradinaru, V., Mogri, M., Thompson, K.R., Henderson, J.M., Deisseroth, K. Optical deconstruction of parkinsonian neural circuitry. Science, 2009. 324:354–359.
5. Krack, P. [Parkinson's disease: deep brain stimulation]. Rev Neurol (Paris). 2002;158(122):135-141.
6. Rueckert, D., Aljabar, P., Heckemann, R.A., Hajnal, J.V., Hammers, A. Diffeomorphic registration using B-splines. Med Image Comput Comput Assist Interv. 2006;9(Pt 2):702-9.
7. Frey, S., Pandya, D.N., Chakravarty, M.M., Bailey, L., Petrides, M., Collins, D.L. An MRI based average macaque monkey stereotaxic atlas and space (MNI monkey space), NeuroImage 2011, doi:10.1016/j.neuroimage.2011.01.040.
8. Prima, S., Ourselin, S., Ayache, N. Computation of the Mid-Sagittal Plane in 3D Brain Images. IEEE Transactions on Medical Imaging, 2002. 21(2):122-138.
9. Duclap, D. et al. Connectomist-2.0: a novel diffusion analysis toolbox for BrainVISA. In: Proc ESMRMB 2012. Lisbon, Portugal: Springer; 2012. (abstract #842).
10. Descoteaux, M., Angelino, E., Fitzgibbons, S., Deriche, R. Regularized, fast, and robust analytical Q-ball imaging. Magn Reson Med. 2007 Sep;58(3):497-510.
11. Perrin, M. et al. Fibre tracking in q-ball fields using regularized particle trajectories. In: Inf Process Med Imaging 2005; 19: 52–63.
